# Aerobic training with a self-selected or predetermined intensity on sleep parameters in adolescents with obesity: a randomized clinical trial

**DOI:** 10.5935/1984-0063.20220015

**Published:** 2022

**Authors:** Maria Julia Lyra, Tércio Araújo do Rego Barros, Thiago Ricardo dos Santos Tenório, Willemax dos Santos Gomes, Antônio Henrique Germano-Soares, Ricardo Freitas-Dias, Marco Túlio De-Mello, Marcos André Moura Santos, Wagner Luiz do Prado

**Affiliations:** 1 University of Pernambuco, Associate PostGraduate Program in Physical Education - Recife - Pernambuco - Brazil.; 2 University of Pernambuco, Hebiatrics Program: Health Determinants in Adolescence - Recife - Pernambuco - Brazil.; 3 Faculdade de Comunicação Tecnologia e Turismo de Olinda, Physical Education departament - Olinda - Pernambuco - Brazil.; 4 Federal Institute from Alagoas - Maragogi - Alagoas - Brazil.; 5 Federal University of Pernambuco, Physical Education Post-Graduate Program - Recife - Pernambuco - Brazil.; 6 University of Pernambuco, Department of Physiotherapy - Petrolina - Pernambuco - Brazil.; 7 Federal University of Minas Gerais, Department of Sports Science - Belo Horizonte - Minas Gerais - Brazil.; 8 California State University San Bernardino, Department of Kinesiology - San Bernardino - California - United States.

**Keywords:** Exercise, Obesity, Adolescent, Sleep

## Abstract

**Objectives:**

To compare the effects of self-selected and predetermined intensity on sleep quality and duration, daytime sleepiness, and sleep efficiency of adolescents with obesity after 12 weeks of aerobic training.

**Material and Methods:**

Thirty-seven adolescents (12 girls), 13-18 years old, with obesity (BMI = 95th) were randomized into a predetermined intensity group (PIG), exercise intensity around 60-70% of heart rate reserve; or self-selected intensity group (SIG), the adolescents chose the speed/intensity at the beginning of each session and were able to change it every 5 minutes. The Pittsburgh Sleep Quality Index (PSQI) and Epworth Sleepiness Scale (ESS) were used to assess sleep outcomes.

**Results:**

No differences were observed for PSQI [0.00 ± 2.00 vs 1.38 ± 2.7; p=0.195; d=0.60 (moderate effect)], sleep duration [-0.95 ± 1.2 vs -0.35 ± 1.6; p=0.358; d=0.41 (small effect)], ESS [(2.10 ± 3.9 vs 1.15 ± 4.5; p=0.195; d=0.23 (small effect)], and sleep efficiency [(81.5 ± 24.0 vs 79.4 ± 17.0; p=0.8.14; d=0.10 (trivial effect)] for the PIG and SIG groups, respectively.

**Conclusion:**

Aerobic training at a self- selected or predetermined intensity does not modulate sleep quality, sleep duration and efficiency, and daytime sleepiness, independent of intensity.

## INTRODUCTION

The high prevalence of childhood obesity is one of the major public health problems worldwide^1,2^. The epidemic of obesity in children has occurred in parallel with a reduction in sleep duration ^3,4^. Studies show an association between sleep and obesity^5,6^, leading to a risk of weight gain and obesity during adolescence^7^. Alterations in sleep quantity, quality, daytime sleepiness, and fatigue could be associated with reduced physical activity levels in adolescents with obesity^8^.

Although small, the effects of aerobic training on sleep parameters are reported as beneficial, in particular, sleep quantity and quality^9,10^. Regarding individuals with obesity, the majority of the research has been conducted in adults^8,11,12^, and data on the pediatric population are scarce. Mendelson et al., (2016) examined the impact of exercise training (i.e., cycling, treadmill, rowing, and resistance training) in a sample of adolescent (age: 14.5 ± 1.5 years; body mass index: 34.0 ± 4.7 kg/m^2^) and 20 healthy-weight, on sleep duration, sleep quality, and physical activity. The authors reported increased sleep duration and sleep efficiency in adolescents with obesity.

Of note, adherence to physical activity programs is one of the major challenges for obesity treatment^13^. In this context, a self-selected exercise intensity (SSI) has been proposed as a possible approach strategy to increase adherence^14,15^. Self-selected intensity (SSI)^16^ is characterized by the control of intensity by the participant, he/she being free to choose and adjust the exercise intensity during the training sessions^17^. In addition, in recent years, SSI has gained greater recognition and is being applied in the treatment of obesity^16,17^. Therefore, it is important to investigate whether there is a difference in sleep parameters between aerobic training with a self-selected or predetermined intensity in adolescents with obesity.

Thus, the purpose of this study was to compare the effects of self-selected and predetermined intensity on sleep quality and duration, daytime sleepiness, and sleep efficiency of adolescents with obesity after 12 weeks of aerobic training. Our hypothesis was that both prescription strategies (self-selected and predetermined intensity) would improve sleep parameters in this population.

## MATERIAL AND METHODS

### Trial design

This is a randomized clinical trial with two parallel groups and pre- and post-intervention evaluations. The outcomes presented here were part of a clinical trial that aimed to compare the effects of self-selected intensity and predetermined intensity exercise on health-related quality of life in adolescents with obesity, after 12 weeks of aerobic training (Brazilian Clinical Trials Registry RBR 2Y8F8R/UTN: U1111-1199-1132), the results of which have been previously published^18^.

### Participant screening

Adolescents from both sexes were recruited through local and social media advertisements and included if they met the following criteria: aged 13-18 years, BMI > 95^th^ for age and sex^19^, within Tanner Stages 3-4^20^, reported no restrictions to physical exercise (Physical Activity Questionnaire – PAR-Q), and agreed to participate in a regular exercise program. Exclusion criteria were: individuals with self-reported genetic, metabolic, or endocrine diseases, who were using medications that could modulate the outcomes (e.g., antihypertensive, hypoinsulinemic, or psychotropics), or were pregnant during the intervention.

### Interventions

The adolescents performed the aerobic exercise on a treadmill, three times per week on alternate days, for 12 weeks. The training sessions were conducted in the morning or in the afternoon. And were divided into: five minutes of warm-up at 4.0 km/h, 25-minutes of training in their respective group, and five minutes of cool-down at 4.0 km/h. Adolescents assigned to the PIG trained at 60-70% of heart rate (HR) reserve during the main part of the protocol^18^. Adolescents assigned to the SIG were instructed to choose their training intensity, for which the following verbal instruction was given at the beginning of every session: “select the intensity/speed at which you think you will be able to complete 35 minutes of exercise; however, you can modify the intensity/speed every 5 minutes”. In both groups, during all sessions, adolescents wore a heart rate monitor (Polar Ft4, Polar®, Kempele, Finland). The final assessments were realized in the end of the last exercise session.

### Outcomes

#### Anthropometry

Body mass was measured to the nearest 0.1kg with a digital scale (Welmy® model 160/300, Brazil), and stature was measured to the nearest 0.5cm using a portable stadiometer (Welmy scale, Welmy® model 160/300, Brazil), with the participants barefoot, with feet together, and head in the Frankfurt horizontal plane, wearing light clothing ^21^. BMI was calculated using the standard formula (weight [kg]/height^2^ [m]) and plotted on charts to verify the percentile for age and sex^19^. Tanner’s stages were collected via self-assessments by the adolescents^22^.

### Sleep quality, duration, and efficiency

Sleep quality and duration were assessed using the Pittsburgh Sleep Quality Index (PSQI), which has previously been validated for Brazilian adolescents with high internal consistency (α = 0.83)^23^ and good test-retest reliability (r = 0.85)^24^. The PSQI measures the sleep habits in the previous month and is composed of 19 questions divided into seven domains (i.e., subjective sleep quality, sleep latency, sleep duration, habitual sleep efficiency, sleep disturbance, use of sleeping drugs, and daytime dysfunction). The final score ranges from zero to 21, with high values indicating poor sleep quality or sleep disorder. Total sleep duration was assessed by the fourth question of the PSQI “how many hours of actual sleep do you get at night”. Sleep efficiency is the percentage of time spent asleep while in bed, calculated by dividing the amount of time spent asleep (in minutes) by the total amount of time in bed (in minutes)^25^.

### Daytime sleepiness

The Epworth Sleepiness Scale (ESS) was used to assess the daytime sleepiness of the adolescents^26,27^. The ESS is a self-administered questionnaire that quantifies an individual’s propensity to fall asleep during eight routine situations: 1-Sitting and reading; 2-Watching TV; 3- Sitting, inactive in a public place; 4- As a passenger in a car for an hour without a break; 5- Lying down to rest in the afternoon when circumstances permit; 6- Sitting and talking to someone; 7-Sitting quietly after a lunch without alcohol; 8- In a car, while stopped for a few minutes in the traffic. The ESS final score ranges from zero to 24 points and higher values (≥10 points) indicate excessive daytime sleepiness.

### Randomization and allocation

For the present study, block randomization was used (randomizer.org). Boys and girls were randomized separately, generating the same ratio of girls/boys per group. Researchers were blinded to group assignment.

### Data analysis

The normality and homogeneity of the data were confirmed by the Shapiro-Wilk and Levene’s tests, respectively. Potential interaction factors (i.e., sex-by- PSQI score; ESS score and sleep time; and age-by-PSQI score; ESS score and sleep time) were evaluated using ANOVA models. As no statistically significant interactions were found, all sex and age groups were analyzed together. Intergroup (PIG vs SIG) differences in changes in the PSQI, sleep duration, and ESS scores from pre- to post-intervention were performed through calculating the absolute changes for each score (Δ= post-intervention – pre-intervention), and differences were evaluated using independent sample t-tests. Effect size (Cohen’s d) was used for both groups, PIG and SIG, after the intervention. Cohen’s d effect sizes were classified as trivial (d< 0.20), small (d = 0.20-0.49), moderate (d = 0.50-0.79), or large (d> 0.80) ^28^. An α value of ≤ 0.05 was used to signify statistical significance. Statistical analyses for all exercise data were performed using SPSS® software, version 21.0 (Statistical Package for the Social Sciences), and Graph-Pad Prism version 5.0.

## RESULTS

**Figure 1:**
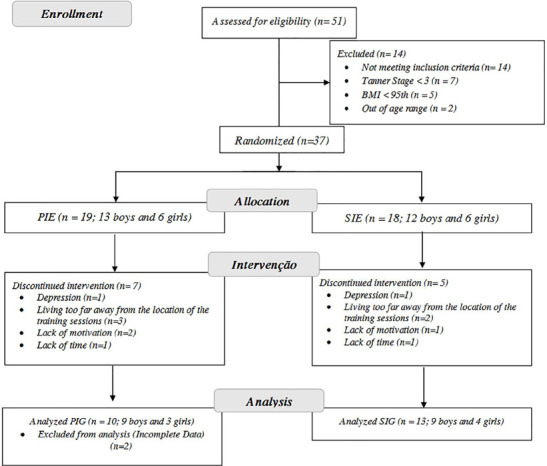
Flow-chart of the study.

The intervention started with 37 adolescents (PIG; n = 19; SIG; n = 18) however, 12 participants dropped out (7 in PIG and 5 in SIG) and were not included in the analyses. The dropouts occurred in the familiarization week (n = 1); 4th-week (n = 1); 5th-week (n = 3); 7th-week (n = 2); and 8th-week (n = 3). The reasons for discontinuing were depression (n = 2); living too far away from the location of the training session (n = 5); lack of motivation (n = 3); and lack of time due to school schedules (n = 2). Two adolescents were excluded from the analysis because they had incomplete sleep data. The flowchart of the study is presented in Figure 1.

The power of the analyzes was calculated (using G*Power v.3.1®), taking into account the sample size at the end of the intervention, and the effect size for the primary outcome variable (PSQI /d=0.60), and the value found for power was 0.99

No baseline differences were observed for age, anthropometry, sleepiness, and sleep efficiency except for the PSQI for which the PIG presented higher values than the SIG (p=0.03) (Table 1). Overall adherence to the experimental protocol was 52.6% for the PIG (n = 10) and 72.2% for the SIG (n= 13).

**Table 1:** General anthropometric and sleep characteristics of the adolescents included in this study.

	PIG (n=10)	SIG (n=13)	P-value
**Age (years)**	14.3 ± 1.8	15.6 ±1.5	0.07
**Body Mass (kg)**	100.7 ± 21.8	98.9 ± 24.9	0.82
**Height (m)**	1.62 ± 0.08	1.64 ± 0.08	0.60
**BMI (kg/m^2^)**	37.27 ± 7.15	37.44± 7.24	0.94
**Pittsburgh Sleep Quality Index**	5.60 ± 3.30	4.92 ± 3.32	0.03
**Epworth Sleepiness Scale**	7.40 ± 3.59	7.07 ± 4.09	0.92
**Sleep Duration (hours)**	8.08 ± 1.44	6.91± 1.38	0.19
**Sleep efficiency (score)**	80.3 ± 23.1	84.5 ± 17.2	0.65

Data presented as mean and standard deviation. BMI: body mass index; PIG: Predetermined Intensity Group and SIG: Self-Selected Intensity Group.

After 12-weeks of aerobic training, no differences were observed for PSQI [0.00 ± 2.00 vs 1.38 ± 2.7; p=0.195; d=0.60], sleep duration [-0.95 ± 1.2 vs -0.35 ± 1.6; p=0.358; d=0.41], ESS [2.10 ± 3.9 vs 1.15 ± 4.5; p=0.195; d=0.23], and sleep efficiency [(81.5 ± 24.0 vs 79.4 ± 17.0; p=0.860; d=0.10] scores for the PIG and SIG groups, respectively. However, the determination of Cohen’s d comparing the PIG and SIG groups post intervention showed trivial to moderate effect sizes for the variables of sleep parameters examined.

**Figure 2:**
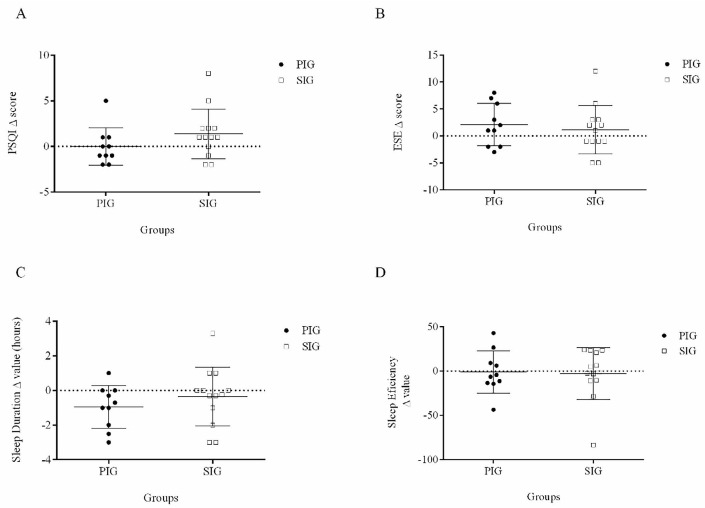
Individual variation in each group after 12 weeks of aerobic exercise at a self-selected (SIG) and predetermined intensity (PIG): PSQI (Panel A), daytime sleepiness (ESE) (Panel B), sleep duration (Panel C), and sleep efficiency (Panel D).

The individual analyses are presented in figure 2. From the total sample of adolescents that concluded the experimental design, 8 (~34.7%) participants of both groups (PIG and SIG) presented reductions of 1 to 2 points (i.e., 4.76% to 9.52% concerning the final score, respectively) in the PSQI (Panel A), whilst 9 (~39.1%) presented reductions of 1 to 5 (i.e., 4.16% to 20.8%, respectively) points in the daytime sleepiness score (Panel B). Moreover, the majority of the obese adolescents (i.e., PIG and SIG) demonstrated a reduction in their sleep duration after the training (Panel C). In this scenario, it is important to highlight that regardless of the reduction in total sleep time, no differences were observed in the sleep efficiency of obese adolescents and the individual analyses showed that most of the adolescents (~86.9%) scored between zero and 2 for sleep efficiency (i.e., > 85% to 65% of efficiency), which represents good efficiency of sleep (Panel D).

## DISCUSSION

This is perhaps the first randomized clinical trial to compare the effects of aerobic training with predetermined versus self-selected intensity on sleep parameters of adolescents with obesity. We hypothesized that aerobic exercise, regardless of being at a self-selected or predetermined intensity, would affect sleep parameters in adolescents with obesity. However, the main results indicated that 12-weeks of aerobic training with a predetermined or self-selected intensity did not influence sleep parameters. Nonetheless, when we observed the effect size and individual responses averages our results are clinically relevant because obesity has a negative effect on sleep quality.

Previous studies have examined the effects of exercise (either timing or intensity) on sleep quality and have found mixed results^10,29^. As it is possible to observe although there are sometimes individuals who range significantly different, the general trend is that the group as a whole remains similar, resulting in non-significance.

Adolescence and obesity are associated with physiological and behavioral changes that affect sleep patterns, especially sleep quantity and quality^30,31^, however, the regular practice of exercise by normal-weight adolescents is associated with a perception of good sleep quality^32^. Previous reviews^33,34^, showing the impact of exercise on sleep have been most frequently expressed in terms of post-exercise changes in sleep-stages, with increases in levels of exercise leading to greater duration and intensity of stage 3 sleep. Despite the certainty about the beneficial small effects of exercise in other populations^10^, studies exploring the role of exercise in adolescents with obesity (body mass index: 34.0 ± 4.7 kg/ m^2^) are scarce, with only one found to date^8^.

In the present study, the PIG performed training exclusively between 60%–70% of their HR reserve, and both groups exercised at 60-80% of their maximum HR, as has been previously published elsewhere^18^. Heart rate variability is just one of the physiological responses to exercise that often presents changes and has been shown to have a direct effect on sleep quality ^35^. However, in the present study, it was observed that both groups worked at the same intensity. In a study by Vincent, Barnett (2017)^36^, light, moderate, and vigorous-intensity physical activity were measured by accelerometers in primary school-aged children. No significant associations were observed between time in bed, total sleep time, and sleep efficiency in any of the directions. Although that study had similar results to the current study, it was performed with school-aged children, whereas the present study was performed with adolescents with obesity.

In a multidisciplinary context, aerobic training has been shown to be an effective and safe feature in the management of obesity and metabolic risks^37,38^. However, these adjustments appear to be dependent on the intensity of the physical effort performed. In adolescents, the results of earlier studies appear contradictory and do not present a consensus, as some report better results in response to exercise at low-intensity^39,40^, others at high intensity^35,37^, and some report no differences in effects between the intensities on the outcome variable^38,41^.

In the present study, although no significant differences were observed between groups, the adherence to the experimental protocol was 62.1 %, representing moderate adherence to the protocol and this fact could increase the range of variation within the sample and influence better comparisons. However, from a clinical point of view, the aerobic training in the PIG and SIG was effective in promoting a reduction in PSQI and daytime sleepiness scores, when considering the individual responses.

Although both groups showed a reduction in sleep time duration, sleep efficiency did not change significantly. A sleep efficiency of 85 percent or higher is considered to be normal, while a sleep efficiency anywhere above 90 percent is considered to be very good ^25^. A sleep efficiency lower than 85 percent is considered poor and is a sign that an individual needs to get more efficient sleep. In hindsight, this might have been due to the other academic and social demands on these adolescents during the period of the intervention. It is important to highlight that, all of our experimental interventions were conducted between August and December; however, we recognize there could be some very important differences in this period of the year because most of the participants were sitting their final school exams, which may lead to changes in the routine of sleep.

Multiple studies have evaluated whether maintaining an exercise training regimen for a sustained duration of time (e.g., 12 weeks) improves sleep. Unfortunately, similar to studies focused on acute exercise and sleep, until recently most exercise training studies focused on individuals with relatively normal sleep patterns at baseline; as one might expect, these studies demonstrated only mild, if any, sleep improvements following exercise training^42^. In addition, among exercise characteristics, longer durations of individual exercise bouts resulted in greater reductions in sleep onset latency and greater adherence to the exercise training regimen was associated with greater improvement in sleep quality, whereas a longer duration of the exercise regimen was associated with smaller improvement in total sleep time^10^.

However, interestingly, protocols of aerobic exercise at moderate intensity did not increase sleep duration or sleep quality in overweight and obese adults which is in agreement with the results of our research^11,43^. For instance, Kjeldsen et al. (2012) observed that only adults with obesity (i.e., BMI: 25-30 kg/m2 , body fat >25%) submitted to high daily doses of aerobic exercise (600 kcal/day) presented increased sleep duration after 13 weeks, and moderate doses (300 kcal/day) did not alter sleep duration or quality. Similarly, Quist et al. (2019), compared groups of overweight and obese (i.e., body mass index: 25-35 kg/m^2^), adults at self-selected, moderate (50% VO2 peak-reserve), and vigorous intensity (70% VO2 peak-reserve) and reported that only the group that exercised at the higher intensity presented increased sleep duration, and none of the groups demonstrated improved sleep quality or sleepiness after 12 weeks of intervention. Furthermore, at 12 weeks, sleep quality scores were greater in the vigorous-intensity group compared to moderate and self-selected intensity^11^.

Therefore, there is a possibility that the relationship between aerobic exercise and sleep in individuals with obesity may be affected by greater volumes and intensities of exercise, which would result in greater energy expenditure^11^. The intensity of exercise can play a fundamental role in homeostatic sleep and can lead to metabolic alterations in the brain that can improve the homeostatic regulation^11^. Previous studies with normal-weight adolescents show that high intensities can improve slow-wave sleep^34^. In addition, there is a theoretical model about energy conservation and body restoration, hypothesizing an increased need for sleep following exercise, thus a session leading to higher energy expenditure would have more impact on sleep patterns, such as duration and quality^11^.

The only research submitting adolescents with obesity (body mass index: 34.0 ± 4.7 kg m^2^) to regular exercise exhibited improvements in sleep quality assessed by full night polysomnography and sleep duration recorded by an accelerometer^8^. However, it is worth mentioning that the authors used a protocol involving both aerobic and resistance exercise with a weekly volume of 240 minutes of exercise^8^. Our study used a protocol that comprises only 44% of the weekly volume that Mendelson et al., applied. Thus the improvements in sleep parameters seen by Mendelson et al., may be due to higher energy expenditures^8^.

The findings of the current study may have important practical applications. Adolescents commonly present sleep problems and these problems can be worsened with obesity^44^. The current study demonstrated that 12 weeks of aerobic training do not improve sleep parameters in adolescents with obesity without sleep complaints, regardless of group assignment. It remains unclear why adolescents with obesity do not benefit from aerobic exercise and the current study has some limitations that need to be mentioned. First, physical activity and food habits were not controlled. Second, we used subjective measures for sleep parameters, and therefore, future studies should employ objective measures (e.g. polysomnography) in order to analyze the effects of exercise training on sleep architecture. Third, exercise sessions were conducted both in the morning and in the afternoon and whether the results would differ according to the time of the day deserves further investigation^12^. Although we had losses in the sample, the comparisons made did not differ significantly (p>0.05) from those considered in our analytical sample, and the losses occurred before the experimental protocol started, so a convenience sample was used. Certainly, these will be the objectives of a future study when we will have data from more volunteers available.

These results, although not statistically significant, provide evidence that working with predetermined or self-selected intensity in adolescents with obesity may be beneficial to better sleep quality, mainly when we consider the individual responses. However, as the slight differences are not statistically significant, the research results prove that regardless of predetermined or self-selected intensity, many parameters of sleep remain similar.

## CONCLUSION

In summary, the present study suggests that either self-selected or predetermined intensity of exercise does not modify the sleep quality, sleep duration, sleep efficiency, or daytime sleepiness of adolescents.
